# Role of the receptor for advanced glycation endproducts (RAGE) in retinal vasodegenerative pathology during diabetes in mice

**DOI:** 10.1007/s00125-015-3523-x

**Published:** 2015-02-17

**Authors:** Carmel M. McVicar, Micheal Ward, Liza M. Colhoun, Jasenka Guduric-Fuchs, Angelika Bierhaus, Thomas Fleming, Andreas Schlotterer, Matthias Kolibabka, Hans-Peter Hammes, Mei Chen, Alan W. Stitt

**Affiliations:** 1 Centre for Experimental Medicine, Queen’s University Belfast, Belfast, BT12 6BA Northern Ireland UK; 2 Department of Medicine and Clinical Chemistry, University of Heidelberg, Heidelberg, Germany

**Keywords:** Acellular capillaries, *Ager*, Diabetic retinopathy, Glucose, Glyoxalase I, HbA_1c_, Leakage, Methylglyoxal, Microglia, Mouse, *Rage*

## Abstract

**Aims/hypothesis:**

The receptor for AGEs (RAGE) is linked to proinflammatory pathology in a range of tissues. The objective of this study was to assess the potential modulatory role of RAGE in diabetic retinopathy.

**Methods:**

Diabetes was induced in wild-type (WT) and *Rage* ^−/−^ mice (also known as *Ager* ^−/−^ mice) using streptozotocin while non-diabetic control mice received saline. For all groups, blood glucose, HbA_1c_ and retinal levels of methylglyoxal (MG) were evaluated up to 24 weeks post diabetes induction. After mice were killed, retinal glia and microglial activation, vasopermeability, leucostasis and degenerative microvasculature changes were determined.

**Results:**

Retinal expression of RAGE in WT diabetic mice was increased after 12 weeks (*p* < 0.01) but not after 24 weeks. *Rage* ^−/−^ mice showed comparable diabetes but accumulated less MG and this corresponded to enhanced activity of the MG-detoxifying enzyme glyoxalase I in their retina when compared with WT mice. Diabetic *Rage* ^−/−^ mice showed significantly less vasopermeability, leucostasis and microglial activation (*p* < 0.05–0.001). *Rage* ^−/−^ mice were also protected against diabetes-related retinal acellular capillary formation (*p* < 0.001) but not against pericyte loss.

**Conclusions/interpretation:**

*Rage* ^−/−^ in diabetic mice is protective against many retinopathic lesions, especially those related to innate immune responses. Inhibition of RAGE could be a therapeutic option to prevent diabetic retinopathy.

**Electronic supplementary material:**

The online version of this article (doi:10.1007/s00125-015-3523-x) contains peer-reviewed but unedited supplementary material, which is available to authorised users.

## Introduction

Diabetic retinopathy is typified by breakdown of the blood–retina barrier (BRB), loss of capillary pericytes and endothelium, microaneurysm formation, neuronal/glial dysfunction and progressive ischaemia in the early (non-proliferative) stages [1]. The pathogenesis of diabetic retinopathy is complex and while the underpinning mechanism(s) remains ill-defined, there is strong evidence that inflammation plays an important role [2]. Even in the early stages of disease inflammatory pathology manifests as activation of resident and infiltrating immune cells, abnormal expression of pro-inflammatory cytokines, upregulation of adhesion molecules and leucostasis in retinal capillaries [2]. This relatively non-specific and sustained response to diabetes-related cell injury seems to be driven, at least in part, by microglia and Müller glia acting as the main resident innate immune cells of the retina.

Innate immune responses are regulated by pattern-recognition receptors (PRRs) expressed by immunologically active cells. PRRs, such as the Toll-like receptors (TLRs), CD36 and the receptor for AGEs (RAGE), can orchestrate innate responses such as cytokine release and immune cell activation [3]. Although the complex interplay of PRRs in the diabetic retina remains poorly understood there has been recent attention given to RAGE and its role in diabetic retinopathy [4–6].

Amongst the major ligands for RAGE are high-mobility group box-1 (HMGB-1), amyloid-β, S100B and AGEs [7], many of which occur in the diabetic retina [8, 9]. Inflammation-related pathways involving RAGE are active in retinal endothelial cells [10] and Müller glia [6] and expression of the receptor is enhanced under diabetic conditions [6]. RAGE signalling evokes extracellular signal-regulated kinase 1/2, mitogen-activated protein kinases and P38, leading to downstream activation of nuclear factor-κB and induction of pro-inflammatory cytokines and/or oxidative stress [11]. Furthermore, blockade of RAGE ligand–receptor binding using a soluble protein fragment (sRAGE) can prevent Müller glial dysfunction [4] during diabetes and retinal capillary leucostasis in AGE-infused normal mice [12]. A recent study by Li et al [5] used a RAGE-inhibiting fusion protein (RAGE-Fc) in diabetic mice and demonstrated a reduced leakage of albumin into neural retina, although there was no significant change in capillary leucostasis. In mice diabetic for 10 months, RAGE blockade prevented acellular capillary formation but not retinal pericyte loss [5]. The current study seeks to investigate retinal lesion progression using mice in which RAGE protein has been deleted and we provide insight into the role RAGE plays in diabetic retinopathy.

## Methods

### Diabetes induction in mice

The *Rage*-knockout mouse (*Rage* ^−/−^; also known as *Ager* ^−/−^) was generated on an SVEV129×C57BL/6 background and backcrossed to C57BL/6 mice for five generations [13]. Wild-type (WT) C57BL/6 mice were purchased from Harlan Laboratories (Bicester, UK) and maintained within the Biological Research Unit at Queen’s University Belfast.

All experiments conformed to the Principles of Laboratory Animal Care (National Institutes of Health, Bethesda, MD, USA) and to the UK Home Office regulations. Diabetes was induced in male *Rage* ^−/−^ mice and WT mice at 12 weeks of age using five daily i.p. injections of streptozotocin (STZ) (Sigma, Gillingham, UK) (50 mg/kg in 0.1 mol/l citrate buffer pH 4.6). Control groups received citrate buffer. Hyperglycaemia was confirmed 10 days after injection using glucometric analysis of tail-prick blood samples (FreeStyle Lite; Abbott Laboratories, Dublin, Ireland). Non-fasting blood glucose concentrations > 13 mmol/l were considered to indicate diabetes. Blood glucose and weight was monitored bi-weekly. HbA_1c_ levels were determined using Glyco-Tek Affinity Column (Helena Biosciences Europe, Gateshead, UK) whenever mice were killed.

### Glyoxalase I activity and methylglyoxal formation in the diabetic retina

Glyoxalase I (GLO-1) enzymatic activity in retinal homogenates from the 4-week groups was assayed using previously published protocols [14]. At 8 and 24 weeks diabetes, the synthesis and purification of methylglyoxal (MG), as well as the synthesis of the derivatising agent and the standards for determination of MG, were prepared according to published procedures [14]. The concentration of MG was determined in tissue as nmol/mg and by derivatisation with 1,2-diamino-4,5-dimethoxybenzene. The protein concentration of the tissue homogenate was determined by the Bradford assay using BSA as a standard.

### GLO-1 and glutathione immunostaining

GLO-1 and glutathione (GSH) immunostaining was assessed using rat anti-GLO-1 (1:200; Abcam, Cambridge, UK) and rabbit anti-GSH (1:1,000; Agrisera, Vännäs, Sweden) on frozen retinal sections (20 μm). The GLO-1 and GSH were visualised using goat anti-rat antibody labelled with Alexa Fluor 488 and goat anti-rabbit Alexa Fluor 568 (Invitrogen, Paisley, UK).

### Quantitative RT-PCR for WT retina

Total RNA of retina was extracted using RNeasy Mini Kit (Qiagen, UK). The level of *Rage* and *Glo-1* (*Glo1*) expression in the retina was examined using quantitative RT-PCR (qPCR) as previously reported [15]. β-Actin was used as the housekeeping gene. Primer sequences are listed in electronic supplementary material (ESM) Table 1.

### Assessment of the BRB

Assessment of the BRB was conducted in cryosections, by assessing albumin leakage from the retinal vasculature. The vasculature was visualised using biotinylated isolectin GS-IB4 (Sigma) followed by binding to streptavidin–Alexa Fluor 488 (Life Technologies, Paisley, UK). Albumin was localised using a goat anti-mouse albumin antibody (Bethyl Laboratories, Montgomery, TX, USA) followed with anti-goat Alexa Fluor 568 (1:500; Life Technologies). Vessel or neuropile-localised fluorescence was visualised by using a Nikon TE EZ-C1 confocal system (Nikon, Kingston Upon Thames, UK). Using NIS Elements software (Nikon) a threshold value of 12,000 was set for measuring image intensity/brightness. The area (μm^2^) of the albumin staining was measured with the 12,000 brightness intensity threshold in the retinal sections. Images were taken at three separate points on the central retina at magnification ×40 and *n* = 5 per group of mice.

### Leucostasis in the retinal vasculature

Leucostasis in diabetic mice was assessed after 4 weeks of diabetes using fluorophore-conjugated concanavalin-A, as described previously [16]. The retinas were mounted on slides and visualised using a Nikon TE EZ-C1 confocal system (Nikon). Two z stacks of the retina for each artery/vein with the capillaries (central and peripheral) were taken and the total number of leucocytes in the arteries, veins and capillaries were counted.

### Assessment of retinal glia and microglia

Microglial cells were identified using rat anti-mouse F4/80 antigen (1:50; AbD Serotec, Kidlington, UK) on retinal flat mounts. The state of microglial activation was ascertained using previously published methods [17]. Müller cell activation was assessed by staining glial fibrillary acidic protein (GFAP) (1:500; Rabbit anti-GFAP; Dako) on retinal sections from mice that had been diabetic for 24 weeks.

### Analysis of retinal DNA damage

DNA damage in the retina was determined by TdT-mediated dUTP-X nick end labelling (TUNEL) assay using the In Situ Cell Death Detection Kit (fluorescein; Roche Applied Science, Penzberg, Germany) according to the manufacturer’s instructions [15]. Positive cells were assessed by image analysis in multiple sections. Images were taken at three separate points on the central retina at magnification ×40 and presented as the average nuclei in the ganglion cell layer. The slides were viewed using epifluorescent microscopy and analysed using NIS Elements (Nikon).

### Quantification of capillary degeneration

Retinal flat mounts were prepared for immunofluorescence evaluation as previously described [15]. Briefly, retinas were stained with biotinylated isolectin GS-IB4 (Sigma) and rabbit anti-mouse collagen IV (Col IV) (1:50; AbD Serotec) overnight and imaged using confocal microscopy (Nikon NIS Elements Basic Research (BR) Version 3.0, TE EZ-C1 confocal system). Four regions were taken at magnification ×40 in the central and peripheral retina. Acellular capillaries < 1.3 μm were assessed in this study, as in our previous study [15]. Retinal pericytes in the various mouse groups were assessed using the trypsin digest technique as previously described [18].

### Graphical presentation and statistical analysis

Statistical analysis and graphical presentation was carried out using Prism 5.0 software (Prism 5, San Diego, CA, USA). One-way ANOVA was conducted to compare overall differences among multiple groups and post hoc comparisons were performed using Tukey’s or Bonferroni’s test. A *p* value of < 0.05 was deemed statistically significant.

## Results

### Characterisation of diabetes

STZ induced a consistent state of diabetes in experimental mice. Analysis of body weight revealed a 14% and 37% reduction in WT and *Rage* ^−/−^ diabetic mice compared with age-matched non-diabetic controls at 24 weeks of diabetes (ESM Fig. 1 a, b). HbA_1c_ showed an approximately twofold increase (at 24 weeks) in diabetic mice compared with non-diabetic mice (*p* < 0.001; ESM Fig. 1c). Blood glucose was significantly increased in the diabetic mice of both WT and *Rage* ^−/−^ mice at 24 weeks of diabetes (ESM Fig. 1d).

### MG and its detoxification in the diabetic retina

**Fig. 1:**
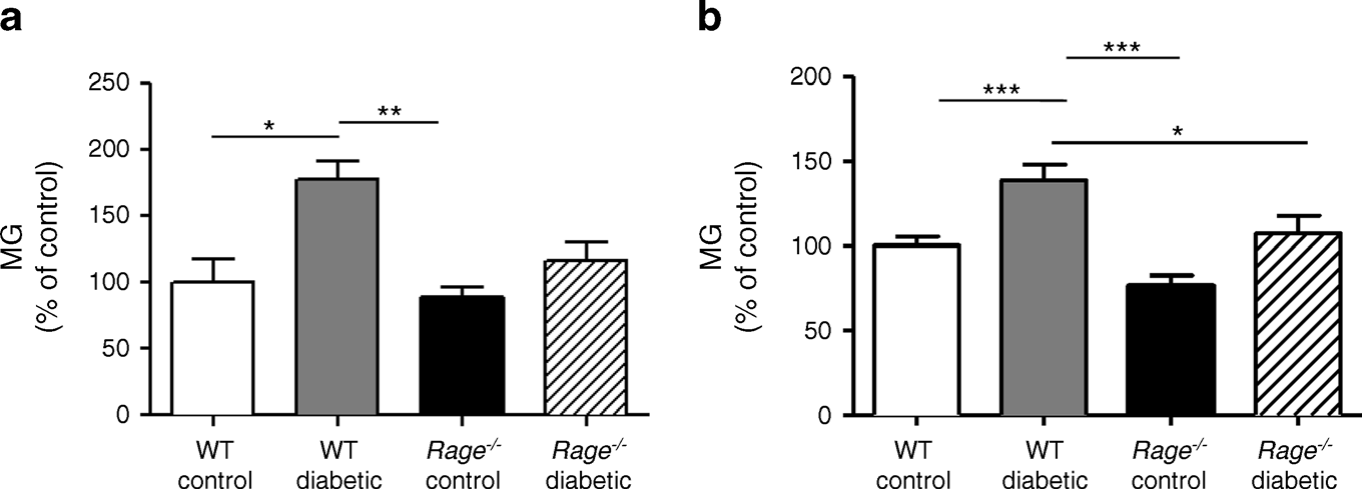
Accumulation of MG-derived adducts in diabetic mouse retina. MG levels were increased in the retina after both 8 (**a**) and 24 (**b**) weeks of diabetes in WT mice. Levels were determined as nmol/mg and presented as a percentage of control. Data are expressed as mean ± SEM (*n* = 3 per group). *p < 0.05, **p < 0.01 and ***p < 0.001 for indicated comparisons

Compared with non-diabetic WT controls, *Rage* mRNA expression in the retina was increased after 12 weeks of diabetes (*p <* 0.01) but this differential was not apparent at 24 weeks (ESM Fig. 2). Since AGEs are key ligands for RAGE in the diabetic retina [9] and MG-derived adducts are the most abundant in this tissue during diabetes [19], we examined the accumulation of the reactive AGE precursor MG in the retina and also the activity of its detoxifying enzyme GLO-1. Examination of retinal homogenates showed that after both 8 and 24 weeks of diabetes, WT diabetic mice had significantly higher levels of MG in the retina compared with non-diabetic controls; this elevation was not apparent in *Rage* ^−/−^ diabetic mice (Fig. 1 a, b).

**Fig. 2:**
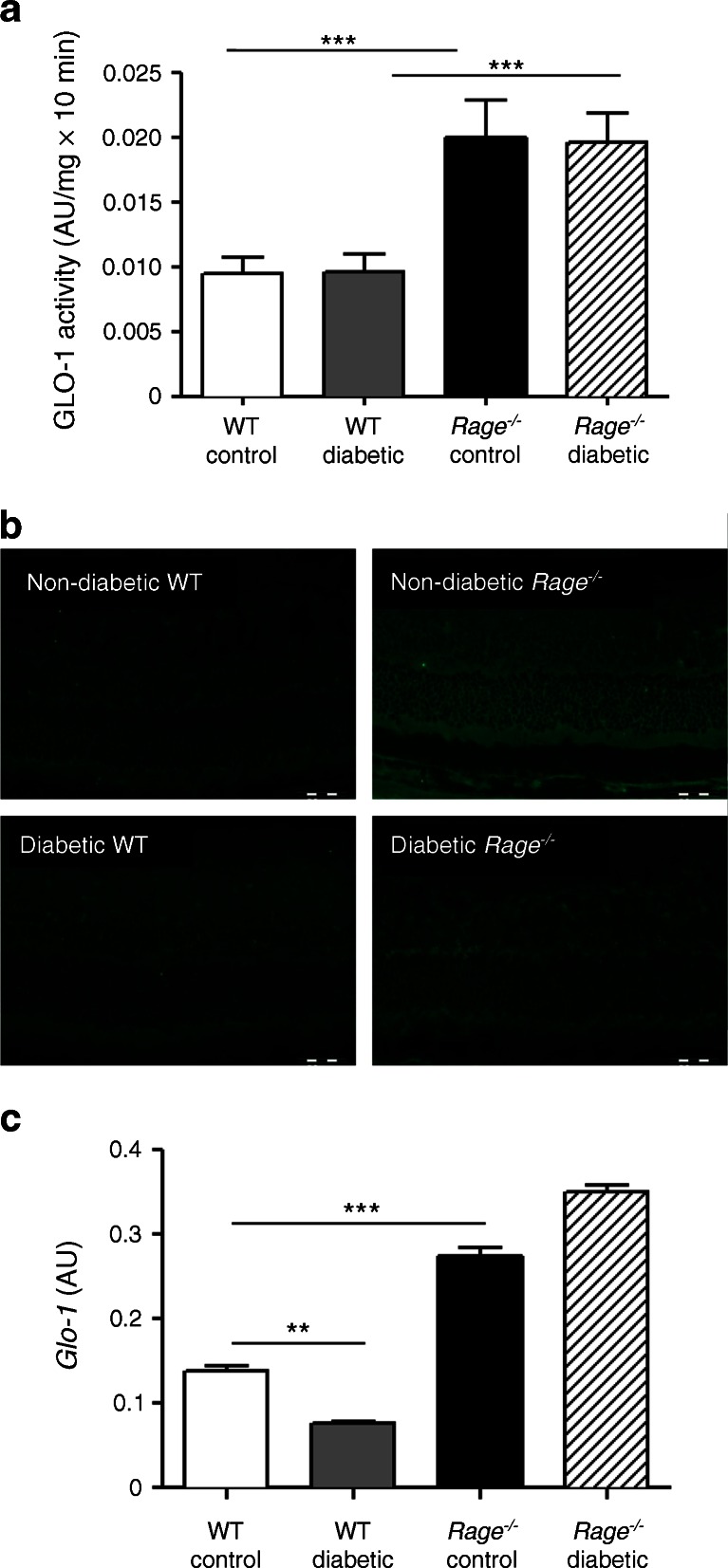
GLO-1 enzymatic activity in diabetic mouse retina. (**a**) GLO-1 enzymatic activity is shown in arbitrary units (AU)/mg × 10 min. (**b**) GLO-1 immunoreactivity was elevated in non-diabetic *Rage* ^−/−^ and diabetic *Rage* ^−/−^ mouse retina compared with the low expression in WT control and WT diabetic mouse retina. (**c**) Retinal *Glo-1* mRNA expression (normalised vs β-actin) in WT diabetic mouse retina was lower than in the WT control and expression in *Rage* ^−/−^ control mouse retina was higher. Data are expressed as mean ± SEM (*n* = 5 per group). **p < 0.01 and ***p < 0.001 for the indicated comparisons

Retinal GLO-1 enzymatic activity and mRNA expression for different durations of diabetes was examined. At 4 weeks, retinal GLO-1 activity was significantly enhanced in *Rage* ^−/−^ mice, irrespective of the presence of diabetes (Fig. 2a). Not only was GLO-1 activity increased, but also immunofluorescent staining suggested that the expression level of GLO-1 was increased at 8 weeks in *Rage* ^−/−^ mouse retinas (Fig. 2b). The transcript of *Glo-1* was also increased significantly at 12 weeks in *Rage* ^−/−^ mouse retinas (Fig. 2c).

Since reduced GSH is required as a co-factor for GLO-1 during detoxification of MG [20], we examined the level of reduced GSH in the retinas. Immunofluorescent staining revealed that reduced GSH occurred at higher levels in the retina of *Rage* ^−/−^ mice when compared with WT counterparts, irrespective of the presence of diabetes (ESM Fig. 3).

### Rage deletion protects against retinal vasopermeability

**Fig. 3:**
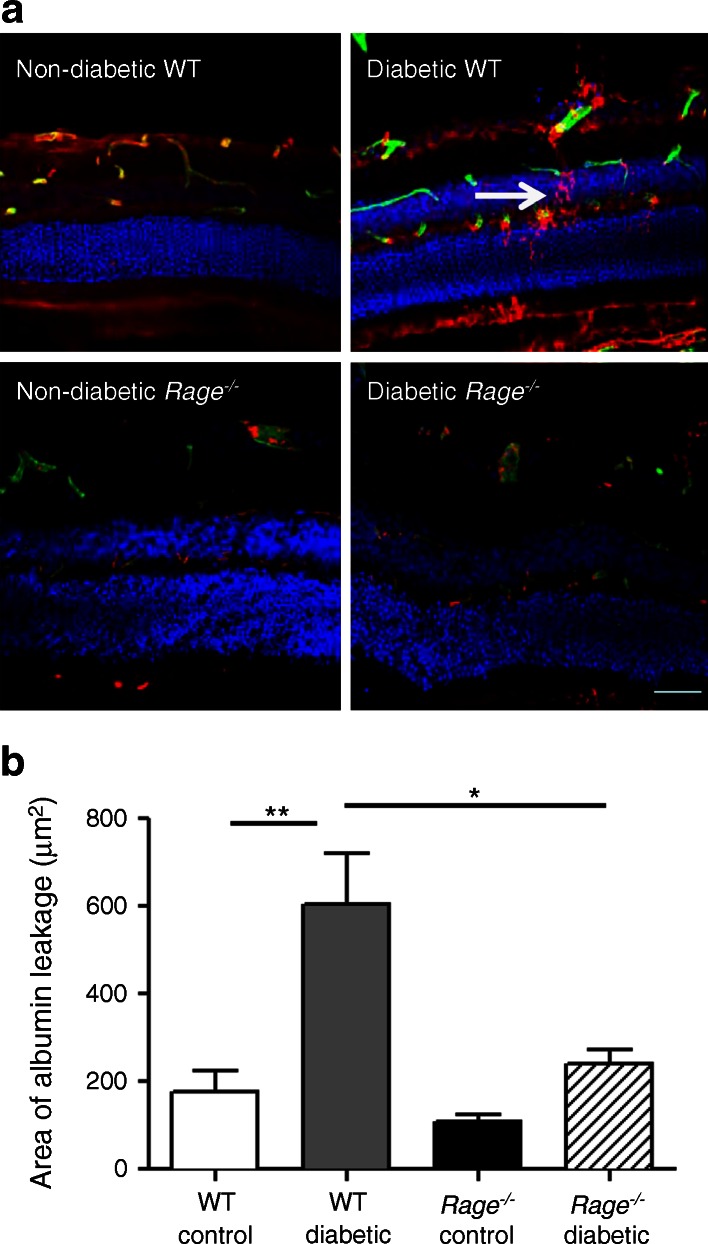
Vasopermeability in diabetic mouse retina is modulated by *Rage*. (**a**) Blood vessel leakage was assessed by albumin (red) staining after 4 weeks of diabetes. There was a significant increase in albumin that had leaked from the blood vessels (stained green with isolectin B4) in WT diabetic mice compared with the control WT mice (white arrow showing leakage). (**b**) Graph of albumin leakage (area in μm^2^) from the retinal blood vessels of *Rage* ^−/−^ diabetic mice showed a decrease in the amount of leakage compared with that in WT diabetic mice. Data are expressed as mean ± SEM (*n* = 6). *p < 0.05 and **p < 0.01 for the indicated comparisons

Vasopermeability was assessed 4 weeks after the induction of diabetes. This time point was chosen since a significant breakdown of the inner BRB in parallel with capillary leucostasis has been shown in this acute phase [21, 22]. Vasopermeability was assessed through visualisation of albumin that had extravasated and was localised in the neural retina. Albumin remained localised to the vessel lumens of the retinal circulation and beneath the retinal pigment epithelium in non-diabetic WT mice (Fig. 3a). In contrast to their non-diabetic counterparts, diabetic WT mice showed significantly greater areas of focal leakage (*p* < 0.01) (Fig. 3 a, b). Diabetic *Rage* ^−/−^ mice appeared to be protected against albumin leakage into the neural retina since there was no significant difference in area of leakage compared with non-diabetic *Rage* ^−/−^ mice (Fig. 3b).

### Regulatory role of RAGE in retinal vascular leucostasis

**Fig. 4:**
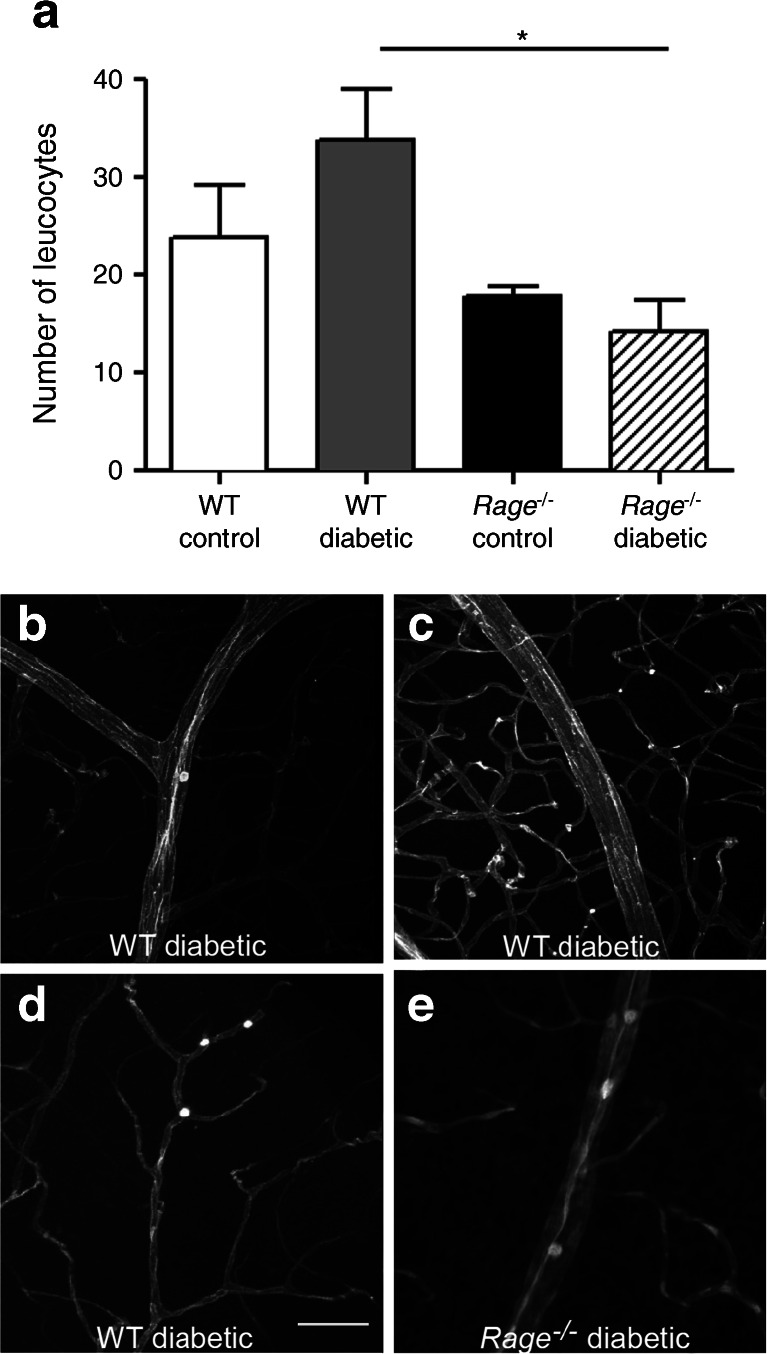
Role of *Rage* deletion in retinal vascular leucostasis during diabetes. (**a**) Leucostasis was assessed in the mouse retina after 4 weeks of diabetes covering 0.1 mm^2^ as the mean of 16 z stacks per retina. Data are expressed as mean ± SEM (*n* = 6). *p < 0.05 for indicated comparison. Leucocytes are shown: at the branches in the vein of a WT diabetic mouse retina (**b**); in the capillaries of a WT diabetic mouse retina (**c**); at the junctions of the capillaries of a WT diabetic mouse retina (**d**) and in the vein of a *Rage* ^−/−^ diabetic mouse retina (**e**). Scale bar, 50 μm

Leucostasis is one of the earliest features in murine diabetic retina and can be detected from as early as 1 week after diabetes induction, persisting for at least 3 months [23]. We studied leucostasis after 4 weeks of diabetes using Concanavalin A–FITC to visualise ‘sticking’ leucocytes inside blood vessels. There was a significant increase in the number of adherent leucocytes in the WT diabetic retina (a response that was apparent for arteries, veins and capillaries) when compared with WT non-diabetic controls (*p* < 0.05) (Fig. 4a). Diabetic *Rage* ^−/−^ mice showed a significant reduction in adherent leucocytes compared with diabetic WT mice (Fig. 4 a, b).

### Rage deletion attenuates diabetes-related glial and neuronal dysfunction

**Fig. 5:**
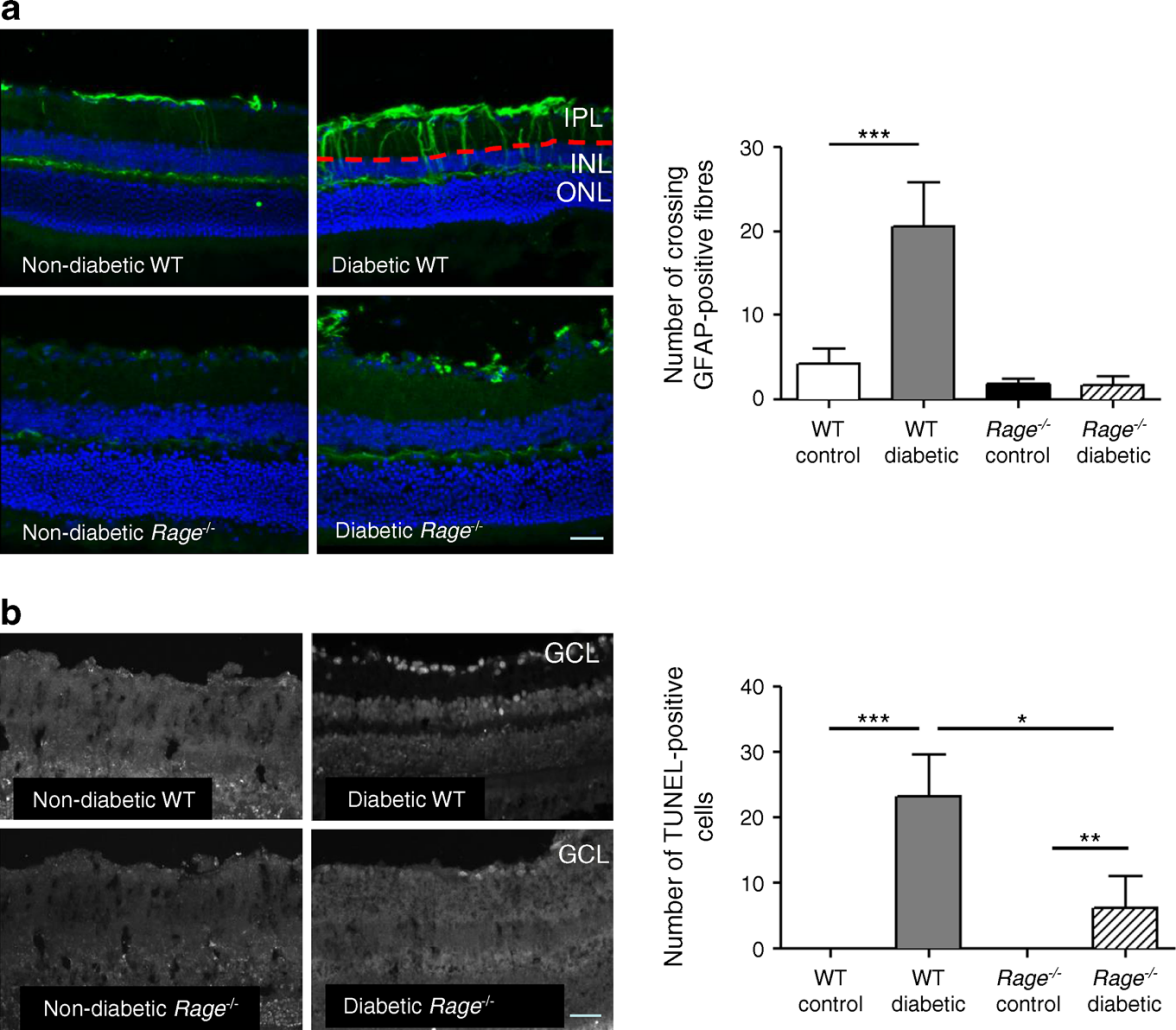
*Rage* deletion alters GFAP expression in retinal Müller glia and DNA strand breaks in ganglion cells. (**a**) Immunostaining of GFAP and graph showing the mean GFAP-positive fibres crossing the inner plexiform layer (IPL) and the inner nuclear layer (INL) in the cohort. The red dotted line indicates the border between the IPL and the INL where the GFAP-positive fibres cross. ONL, outer nuclear layer. (**b**) TUNEL positivity was assessed on the sections of retina from all the experimental groups. GCL, ganglion cell layer. Scale bar, 50 μm. Data are expressed as mean ± SEM (*n* = 4). *p < 0.05, **p < 0.01 and ***p < 0.001 for indicated comparisons

Glial responses were evaluated using the stress-indicator GFAP. In the non-diabetic mouse retina, GFAP was limited in the astrocytes and end feet of Müller cells (Fig. 5a). In diabetic mice GFAP could be detected in the cell processes of Müller cells, indicating Müller cell activation or damage. *Rage* ^−/−^ diabetic mice did not show this typical upregulation of GFAP in the Müller cells (Fig. 5a).

TUNEL-positive cells, indicating DNA strand breaks, were significantly more abundant in the diabetic mice than in non-diabetic controls (*p* < 0.001) (Fig. 5b). TUNEL-positive cells were mainly localised to the ganglion cell layer but they were also present in the inner and outer nuclear layers. In *Rage* ^−/−^ mice, diabetic conditions also increased the number of TUNEL-positive cells, especially in the ganglion cell layer (*p* < 0.01) (Fig. 5b). However, comparison between the WT and *Rage* ^−/−^ diabetic mice showed that the absence of RAGE significantly protected the diabetic retina against DNA breaks (*p* < 0.05) (Fig. 5b).

### RAGE plays a role in microglial activation during diabetic retinopathy

**Fig. 6:**
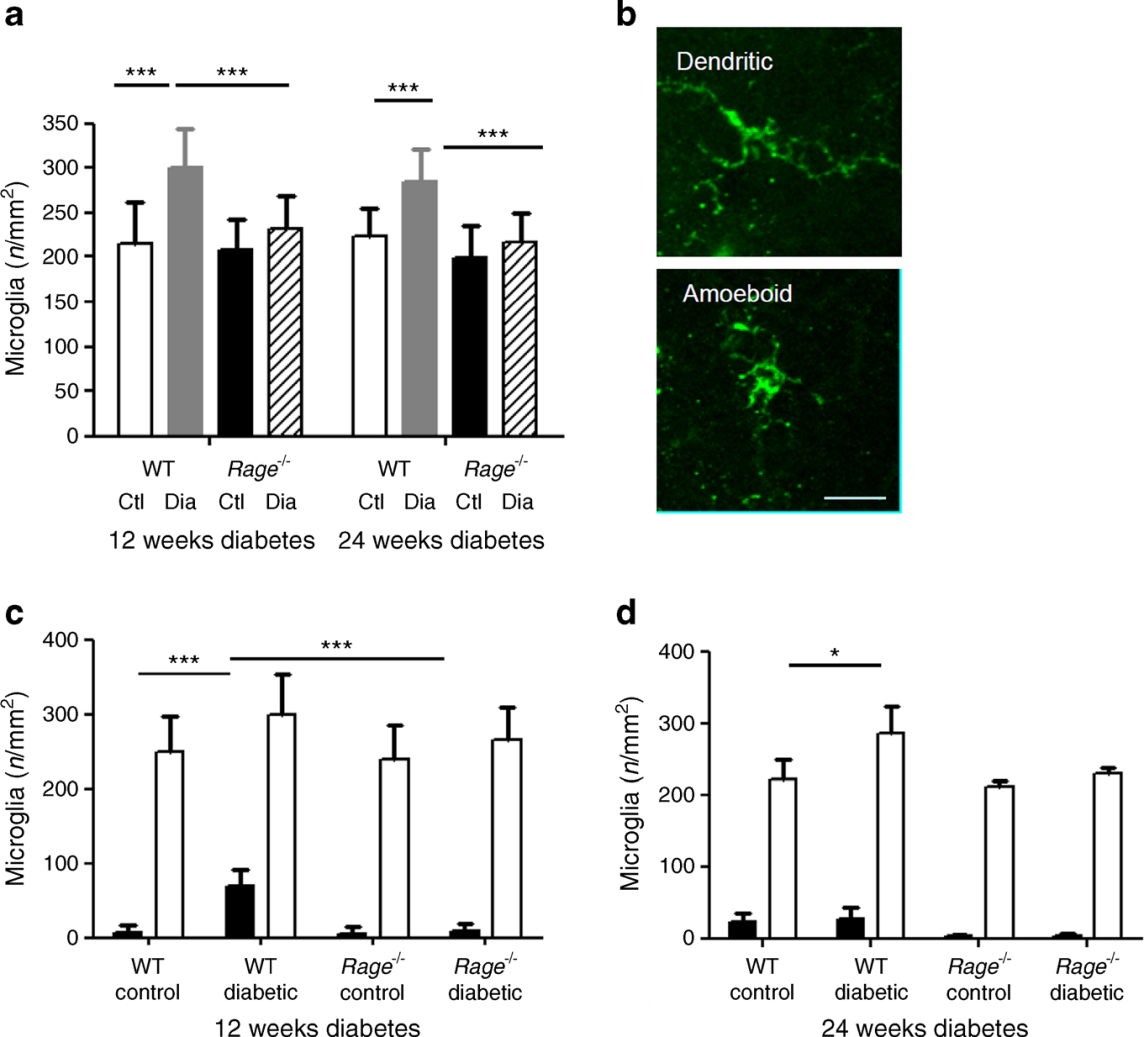
Microgliosis in mouse retinas at 12 and 24 weeks of diabetes. (**a**) Total microglia cell numbers at 12 and 24 weeks of diabetes illustrating more microglia in the WT diabetic mouse than in the other groups. Ctl, control; Dia, diabetic. (**b**) Representative images of dendritic (resting) and amoeboid (active) microglia, taken from *Rage* ^−/−^ control groups. Scale bar, 25 μm. (**c**, **d**) Activation states of microglia were ascertained, post image capture. These graphs illustrate activated (black bars) and resting microglia (white bars) labelled with F4/80 after 12 and 24 weeks of diabetes. Data are expressed as mean ± SEM (*n* = 6). *p < 0.05 and ***p < 0.001 for indicated comparisons

An increase in microglial activation and number of cells in the neuropile has been previously demonstrated in diabetic retinopathy [24]. Following both 12 and 24 weeks diabetes duration in WT mice, there was a significant increase in the numbers of F4/80-positive microglia in the retina when compared with non-diabetic controls (*p* < 0.001) (Fig. 6a). *Rage* ^−/−^ mice did not demonstrate this diabetes-induced increase and, indeed, there were significantly less positive microglia in the retinas of *Rage* ^−/−^ diabetic mice compared with WT diabetic mice (*p* < 0.001) (Fig. 6a).

Microglia presented in various states of activation in normal retina from both WT and *Rage* ^−/−^ mice; however, the ‘classical’ resting (ramified/dendritic; Fig. 6b) state was predominant (Fig. 6 b, c). Following 12 weeks of diabetes, there was a significant increase in activated microglia in the WT diabetic retina whereas this increase was absent in *Rage* ^−/−^ mice (Fig. 6c). After 24 weeks of diabetes, there was no apparent increase in microglial activation compared with non-diabetic controls, only an increase in resting microglia between WT control and WT diabetic (Fig. 6d).

### RAGE deletion protects against diabetes-mediated retinal capillary degeneration

**Fig. 7:**
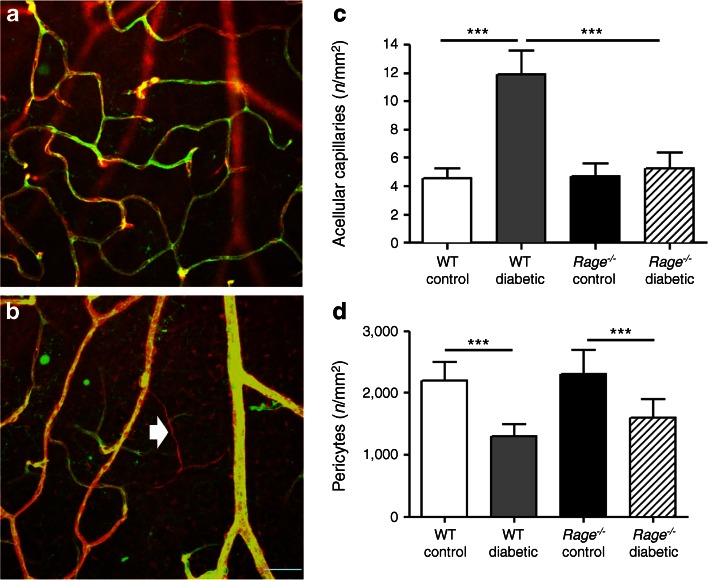
Retinal capillary degeneration and pericyte dropout during diabetes after 24 weeks of diabetes. Flat-mounted retina from WT control and diabetic mice in the deeper plexus (**a**) and the superficial plexus (**b**), illustrating capillaries visualised using isolectin B4 (endothelium; green) and Col IV (basement membrane; red). Acellular capillaries show continuance of Col IV positivity but loss of endothelium (arrow). Scale bar, 50 μm. (**c**) Graph showing the mean number of acellular capillaries in the retina. Data are expressed as mean ± SEM (*n* = 6). ***p < 0.001 for the indicated comparisons. (**d**) Graph showing pericyte coverage in the retina. Data expressed as mean ± SD (*n* = 7). ***p < 0.001 for the indicated comparisons

Acellular capillary formation and the death of pericytes and endothelium in the retina is a hallmark in mice beyond 5 months of diabetes [25]. By definition, acellular capillaries do not have endothelial cells and the vessels are isolectin B4 negative but since a basement membrane persists in these acellular vessels, Col IV immunoreactivity remains present (Fig. 7a, b). When retinal flat mounts were evaluated and quantified for isolectin/Col IV staining, there was a significant increase in acellular capillaries in WT diabetic mice compared with non-diabetic controls (*p* < 0.001) (Fig. 7c). This diabetes-mediated pathology was absent in the retina from *Rage* ^−/−^ diabetic mice. In trypsin-digested preparations, the number of pericytes in the retinal capillaries was significantly reduced in diabetic WT mice compared with controls. Pericyte loss was also significant in the *Rag*e^−/−^ diabetic mice (Fig. 7d).

## Discussion

RAGE plays a significant role in inflammation-linked disorders such as Alzheimer’s disease and atherosclerosis [26, 27]. In the context of diabetic complications, activation of RAGE by a range of ligands has been strongly associated with inflammatory pathology in the kidney and peripheral nerves and vessels [28]. In the current investigation, we show that *Rage* ^−/−^ mice are protected from some of the key early-stage lesions of diabetic retinopathy.

*Rage* deletion had no significant impact on glycaemia (based on HbA_1c_) or characteristic deficits in weight gain. However, diabetes did increase RAGE expression in the murine retina after 12 weeks of diabetes, although this elevation was not maintained up to 24 weeks. RAGE expression is widespread in the retinal neuropile of rats and mice, being especially high in the Müller glia [4], and it has been shown to increase in diabetic rats concomitantly with ligands such as AGEs and S100b [6, 29].

AGEs are ligands for RAGE and many are derived from the α-oxoaldehyde MG, which occurs at high levels in the diabetic retina and gives rise to adducts such as *N* ^ε^-(carboxy-ethyl) lysine and MG-derived hydroimidazolone-1 [30]. An important defence against MG-related toxicity is the glyoxalase complex (formed from GLO-1 and glyoxalase II components), which converts MG to d-lactate using GSH as a co-factor [31]. We have previously reported that overexpression of GLO-1 in diabetic rats protects against retinopathy [30]. The current study now shows that *Rage* ^−/−^ mice possess significantly enhanced retinal GLO-1 activity, a higher GLO-1 expression level and enhanced GSH levels when compared with WT mice. This explains the reduced levels of MG in *Rage* ^−/−^ diabetic mice compared with their WT diabetic counterparts. The relationship between RAGE and GLO-1 during diabetes has been reported previously [32, 33] and it has been suggested that upregulation of the enzyme is linked to RAGE-mediated transcriptional activation of Myd88 [34]. In the current study we have demonstrated that *Rage* ^−/−^ mice have higher levels of retinal GSH than their WT counterparts and this may also account for why GLO-1 activity is also elevated in these mice.

Interactions between circulating immune cells and the retinal vasculature probably begin with leucostasis, which may contribute to capillary occlusion and breakdown of the BRB [35]. An earlier study suggested that neutrophils become less sticky after treatment with RAGE antibody [36]. It is possible that leucocytes from *Rage* ^−/−^ diabetic mice are less sticky due to the absence of this receptor. Deletion of *Rage* in diabetic mice provided protection against vascular leucostasis and vasopermeability and this agrees with the findings of previous studies that used therapeutic approaches. For example, reducing the bioavailability of RAGE ligands (using sRAGE) prevented vasopermeability in mice [12]. Deletion of *Rage* transcription resulted in a marked protection against adhesion of leucocytes. While it is uncertain whether the acute-phase leucostasis phenomenon is a major contributor to capillary degeneration [37], it seems likely that pro-inflammatory processes involving bone marrow-derived cells make a contribution to diabetic retinopathy [38]. Although not evaluated in the current study, there is evidence that TLRs may be involved in retinopathy [39]. The established interplay between TLRs and RAGE is potentially important and could contribute to innate immune responses in the retina, especially since they share common ligand interactions with S100 and HMGB1 [10]. Müller glia act as inflammatory activators and this response is diminished by sRAGE treatment in *db*/*db* mice [4] and, as shown in the current study, genetic deletion of *Rage*. Professional immune cells, which reside in proximity to retinal blood vessels (perivascular macrophages) or within various layers of the neuropile (microglia), are of even more critical importance [40]. While these cells have a homeostatic function, they are also linked to neuroinflammation in the human diabetic retina [24] and animal models [41, 42]. RAGE plays a role in monocyte migration and microglial activation [43, 44] by altering the expression of adhesion molecules, facilitating monocytic extravasation into the CNS and altering microglial cells from resting to activated states [45].

The current study suggests a role for RAGE in microglial activation, especially after 12 weeks of diabetes at which time *Rage* ^−/−^ mice were protected from this response. This bimodal response according to the duration of diabetes may also reflect the altered expression levels of RAGE between 12 and 24 weeks. Further supporting evidence comes from *Rage* ^−/−^ mice subjected to laser-induced choroidal neovascularisation; these mice showed reduced lesion size alongside diminution of immune cell activation when compared with WT controls [46].

In rodent models of diabetic retinopathy, pericyte dropout and acellular capillary formation are ‘gold standard’ markers of retinopathy progression. In the current study, analysis using both trypsin digests and confocal microscopy of whole mounts showed significant vasodegeneration at 24 weeks in WT diabetic mice. *Rage* ^−/−^ mice were protected from diabetes-induced acellular capillary formation, providing strong evidence of an important role for this receptor in diabetic retinopathy. The fact that pericyte loss was not markedly prevented when RAGE was absent is in keeping with studies using RAGE blockade [38] and suggests that loss of these cells is not strongly associated with the RAGE pathway. Our data support the suggestion that inflammatory cascades are linked to retinal vascular damage as diabetes progresses. As a component of the innate immune response, RAGE plays an important role in diabetic retinopathy.

## Electronic supplementary material

Below is the link to the electronic supplementary material.

ESM Fig. 1 (PDF 36.4 kb)

ESM Fig. 2 (PDF 13 kb)

ESM Fig. 3 (PDF 69 kb)

ESM Table 1 (PDF 62 kb)

## Abbreviations

BRB: Blood–retina barrier
Col IV: Collagen IV
GFAP: Glial fibrillary acidic protein
GLO-1: Glyoxalase I
GSH: Glutathione
HMGB-1: High-mobility group box-1
MG: Methylglyoxal
PRR: Pattern-recognition receptor
qPCR: Quantitative RT-PCR
RAGE-Fc: RAGE-inhibiting fusion protein
RAGE: Receptor for AGEs
sRAGE: Soluble protein fragment of RAGE
STZ: Streptozotocin
TUNEL: TdT-mediated dUTP-X nick end labelling
TLR: Toll-like receptor
WT: Wild-type

## References

[1] Stitt AW, Lois N, Medina RJ, Adamson P, Curtis TM (2013). Advances in our understanding of diabetic retinopathy. Clin Sci (Lond).

[2] Tang J, Kern TS (2011). Inflammation in diabetic retinopathy. Prog Retin Eye Res.

[3] Takeuchi O, Akira S (2010). Pattern recognition receptors and inflammation. Cell.

[4] Barile GR, Pachydaki SI, Tari SR (2005). The RAGE axis in early diabetic retinopathy. Invest Ophthalmol Vis Sci.

[5] Li G, Tang J, Du Y, Lee CA, Kern TS (2011). Beneficial effects of a novel RAGE inhibitor on early diabetic retinopathy and tactile allodynia. Mol Vis.

[6] Zong H, Ward M, Madden A (2010). Hyperglycaemia-induced pro-inflammatory responses by retinal Muller glia are regulated by the receptor for advanced glycation end-products (RAGE). Diabetologia.

[7] Barlovic DP, Soro-Paavonen A, Jandeleit-Dahm KA (2011). RAGE biology, atherosclerosis and diabetes. Clin Sci (Lond).

[8] Chen M, Curtis TM, Stitt AW (2013). Advanced glycation end products and diabetic retinopathy. Curr Med Chem.

[9] Zong H, Ward M, Stitt AW (2011). AGEs, RAGE, and diabetic retinopathy. Curr Diab Rep.

[10] Mohammad G, Siddiquei MM, Othman A, Al-Shabrawey M, Abu El-Asrar AM (2013). High-mobility group box-1 protein activates inflammatory signaling pathway components and disrupts retinal vascular-barrier in the diabetic retina. Exp Eye Res.

[11] Bierhaus A, Humpert PM, Stern DM, Arnold B, Nawroth PP (2005). Advanced glycation end product receptor-mediated cellular dysfunction. Ann N Y Acad Sci.

[12] Moore TC, Moore JE, Kaji Y (2003). The role of advanced glycation end products in retinal microvascular leukostasis. Invest Ophthalmol Vis Sci.

[13] Constien R, Forde A, Liliensiek B (2001). Characterization of a novel EGFP reporter mouse to monitor Cre recombination as demonstrated by a Tie2 Cre mouse line. Genesis.

[14] McLellan AC, Phillips SA, Thornalley PJ (1992). The assay of methylglyoxal in biological systems by derivatization with 1,2-diamino-4,5-dimethoxybenzene. Anal Biochem.

[15] McVicar CM, Hamilton R, Colhoun LM (2011). Intervention with an erythropoietin-derived peptide protects against neuroglial and vascular degeneration during diabetic retinopathy. Diabetes.

[16] Chen Y, Hu Y, Moiseyev G, Zhou KK, Chen D, Ma JX (2009). Photoreceptor degeneration and retinal inflammation induced by very low-density lipoprotein receptor deficiency. Microvasc Res.

[17] Pannasch U, Farber K, Nolte C (2006). The potassium channels Kv1.5 and Kv1.3 modulate distinct functions of microglia. Mol Cell Neurosci.

[18] Dietrich N, Hammes HP (2012). Retinal digest preparation: a method to study diabetic retinopathy. Methods Mol Biol.

[19] Karachalias N, Babaei-Jadidi R, Ahmed N, Thornalley PJ (2003). Accumulation of fructosyl-lysine and advanced glycation end products in the kidney, retina and peripheral nerve of streptozotocin-induced diabetic rats. Biochem Soc Trans.

[20] Thornalley PJ (1998). Glutathione-dependent detoxification of alpha-oxoaldehydes by the glyoxalase system: involvement in disease mechanisms and antiproliferative activity of glyoxalase I inhibitors. Chem Biol Interact.

[21] Qaum T, Xu Q, Joussen AM (2001). VEGF-initiated blood-retinal barrier breakdown in early diabetes. Invest Ophthalmol Vis Sci.

[22] Joussen AM, Poulaki V, Tsujikawa A (2002). Suppression of diabetic retinopathy with angiopoietin-1. Am J Pathol.

[23] Ishida S, Usui T, Yamashiro K (2003). VEGF164 is proinflammatory in the diabetic retina. Invest Ophthalmol Vis Sci.

[24] Zeng HY, Green WR, Tso MO (2008). Microglial activation in human diabetic retinopathy. Arch Ophthalmol.

[25] Zheng L, Du Y, Miller C (2007). Critical role of inducible nitric oxide synthase in degeneration of retinal capillaries in mice with streptozotocin-induced diabetes. Diabetologia.

[26] Ramasamy R, Yan SF, Schmidt AM (2009). RAGE: therapeutic target and biomarker of the inflammatory response—the evidence mounts. J Leukoc Biol.

[27] Sorci G, Riuzzi F, Giambanco I, Donato R (2013). RAGE in tissue homeostasis, repair and regeneration. Biochim Biophys Acta.

[28] Ramasamy R, Yan SF, Schmidt AM (2012). The diverse ligand repertoire of the receptor for advanced glycation endproducts and pathways to the complications of diabetes. Vasc Pharmacol.

[29] Curtis TM, Hamilton R, Yong PH (2011). Muller glial dysfunction during diabetic retinopathy in rats is linked to accumulation of advanced glycation end-products and advanced lipoxidation end-products. Diabetologia.

[30] Berner AK, Brouwers O, Pringle R (2012). Protection against methylglyoxal-derived AGEs by regulation of glyoxalase I prevents retinal neuroglial and vasodegenerative pathology. Diabetologia.

[31] Kuhla B, Luth HJ, Haferburg D, Boeck K, Arendt T, Munch G (2005) Methylglyoxal, glyoxal, and their detoxification in Alzheimer’s disease. Ann N Y Acad Sci 1043:211–216 10.1196/annals.1333.026 16037241

[32] Wu F, Feng JZ, Qiu YH (2013). Activation of receptor for advanced glycation end products contributes to aortic remodeling and endothelial dysfunction in sinoaortic denervated rats. Atherosclerosis.

[33] Yao D, Brownlee M (2010). Hyperglycemia-induced reactive oxygen species increase expression of the receptor for advanced glycation end products (RAGE) and RAGE ligands. Diabetes.

[34] Zeng S, Zhang QY, Huang J (2012). Opposing roles of RAGE and Myd88 signaling in extensive liver resection. FASEB J.

[35] Chibber R, Ben-Mahmud BM, Chibber S, Kohner EM (2007). Leukocytes in diabetic retinopathy. Curr Diabetes Rev.

[36] Toure F, Zahm JM, Garnotel R (2008). Receptor for advanced glycation end-products (RAGE) modulates neutrophil adhesion and migration on glycoxidated extracellular matrix. Biochem J.

[37] Gubitosi-Klug RA, Talahalli R, Du Y, Nadler JL, Kern TS (2008). 5-Lipoxygenase, but not 12/15-lipoxygenase, contributes to degeneration of retinal capillaries in a mouse model of diabetic retinopathy. Diabetes.

[38] Li G, Veenstra AA, Talahalli RR (2012). Marrow-derived cells regulate the development of early diabetic retinopathy and tactile allodynia in mice. Diabetes.

[39] Fujimoto T, Sonoda KH, Hijioka K (2010). Choroidal neovascularization enhanced by Chlamydia pneumoniae via Toll-like receptor 2 in the retinal pigment epithelium. Invest Ophthalmol Vis Sci.

[40] Xu H, Chen M, Forrester JV (2009). Para-inflammation in the aging retina. Prog Retin Eye Res.

[41] Barber AJ, Antonetti DA, Kern TS (2005). The Ins2Akita mouse as a model of early retinal complications in diabetes. Invest Ophthalmol Vis Sci.

[42] Rungger-Brandle E, Dosso AA, Leuenberger PM (2000). Glial reactivity, an early feature of diabetic retinopathy. Invest Ophthalmol Vis Sci.

[43] Hughes EH, Schlichtenbrede FC, Murphy CC (2004). Minocycline delays photoreceptor death in the rds mouse through a microglia-independent mechanism. Exp Eye Res.

[44] Bianchi R, Kastrisianaki E, Giambanco I, Donato R (2011). S100B protein stimulates microglia migration via RAGE-dependent up-regulation of chemokine expression and release. J Biol Chem.

[45] Giri R, Shen Y, Stins M (2000). beta-amyloid-induced migration of monocytes across human brain endothelial cells involves RAGE and PECAM-1. Am J Physiol Cell Physiol.

[46] Chen M, Glenn JV, Dasari S (2014). RAGE regulates immune cell infiltration and angiogenesis in choroidal neovascularization. PLoS One.

