# Supplementary material for: Role of the receptor for advanced glycation endproducts (RAGE) in retinal vasodegenerative pathology during diabetes in mice
Source: Diabetologia. 2015 Feb 17;58(5):1129–37. doi: 10.1007/s00125-015-3523-x (PMC4392170; doi:10.1007/s00125-015-3523-x)
Supplement: Supplementary file 1 — (PDF 36.4 kb) [file 125_2015_3523_MOESM1_ESM.pdf]

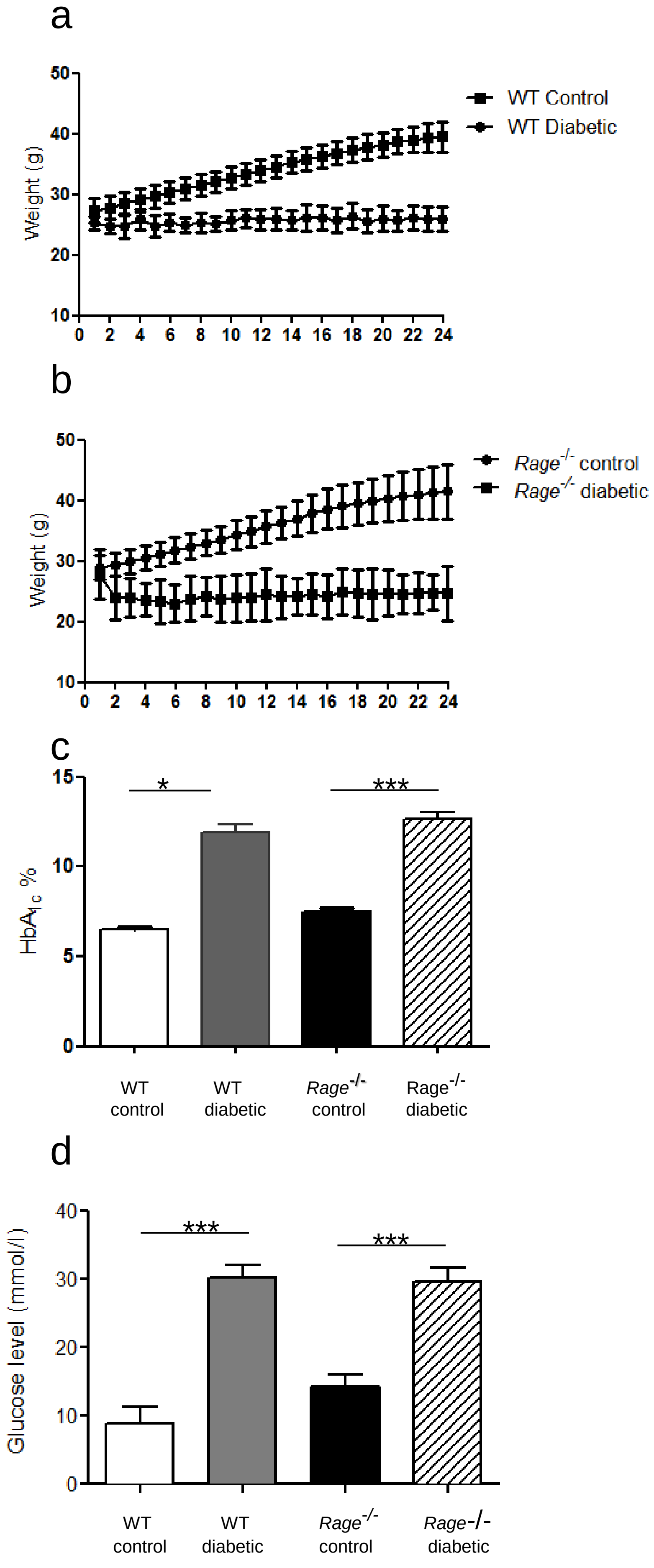
a
Weight (g)
WT Control
WT Diabetic
b
Weight (g)
Rage-/- control
Rage-/- diabetic
c
HbA1c %
*
***
WT control
WT diabetic
Rage-/- control
Rage-/- diabetic
d
Glucose level (mmol/l)
***
***
WT control
WT diabetic
Rage-/- control
Rage-/- diabetic

Figure 1S. Weight and HbA1c levels in control and diabetic mice.
WT diabetic mice show weight loss whereas non-diabetic WT animals continue to increase in weight throughout the study (a). Following diabetes induction, *Rage* $^{-/-}$ mice show a similar pattern of weight loss to that observed in WT animals (b). Terminal cardiac puncture allowed for HbA_{1c} analysis at 24 wks of diabetes (c) and blood glucose at 24 wks is shown in (d) (WT Control; WT Diabetic; *Rage*$^{-/-}$ Control; *Rage*$^{-/-}$ Diabetic n=10). To convert values for HbA_{1c} in DCCT% into mmol/mol, subtract 2.15 and multiply by 10.929. *$p < 0.05$, and ***$p < 0.001$. Data expressed as mean ± SEM.
