# Supplementary material for: Role of the receptor for advanced glycation endproducts (RAGE) in retinal vasodegenerative pathology during diabetes in mice
Source: Diabetologia. 2015 Feb 17;58(5):1129–37. doi: 10.1007/s00125-015-3523-x (PMC4392170; doi:10.1007/s00125-015-3523-x)
Supplement: Supplementary file 2 — (PDF 13 kb) [file 125_2015_3523_MOESM2_ESM.pdf]

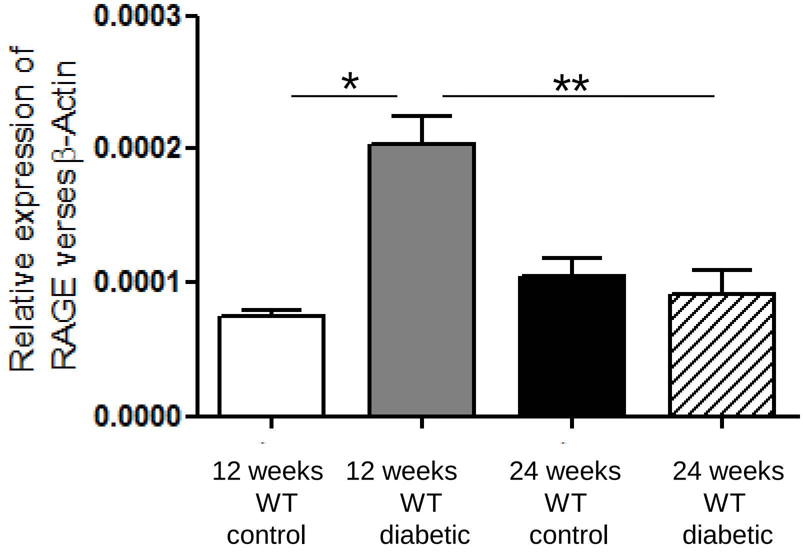

Figure 2S. RAGE expression in diabetic retina

(a) Retinal RAGE mRNA expression is significantly upregulated in WT retina mice after 12 weeks diabetes (DB) in comparison to non-diabetic (ND) controls (* $p<0.05$). This was ameliorated at 24 weeks diabetes (**$p<0.01$) with levels reduced to that of control mice.
