# Supplementary material for: Role of the receptor for advanced glycation endproducts (RAGE) in retinal vasodegenerative pathology during diabetes in mice
Source: Diabetologia. 2015 Feb 17;58(5):1129–37. doi: 10.1007/s00125-015-3523-x (PMC4392170; doi:10.1007/s00125-015-3523-x)
Supplement: Supplementary file 3 — (PDF 69 kb) [file 125_2015_3523_MOESM3_ESM.pdf]

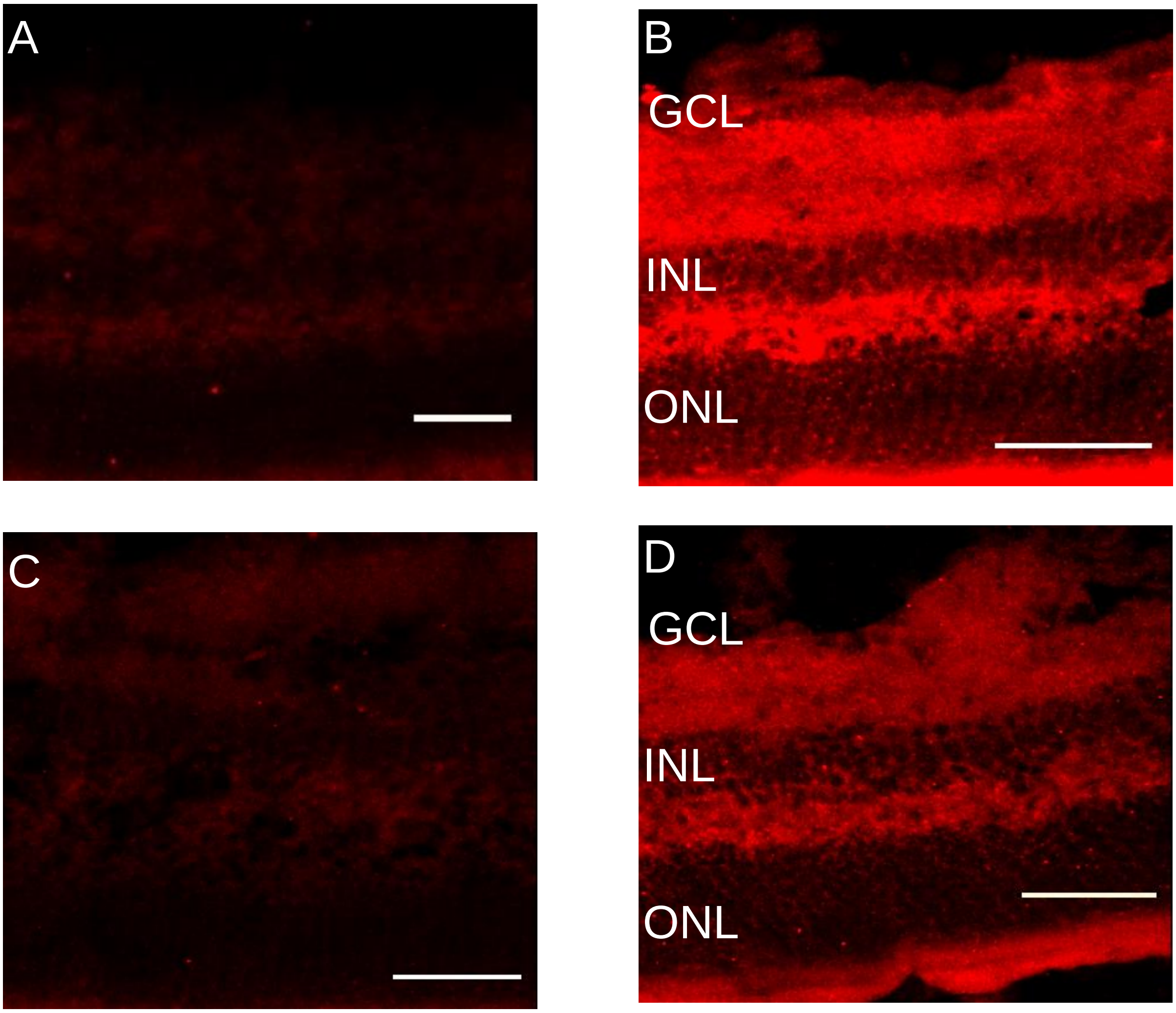

Figure 3S. GSH immunoreactivity in WT and RAGE -/- diabetic retina

(a) GSH was lowly expressed in WT Control (12wks) but protein levels were markedly greater in *Rage*$^{-/-}$ control (12wks) (b). Similarily, GSH occurred at low levels in WT Diabetic (12wks) (c) but was elevated *Rage*$^{-/-}$ Diabetic (12wks) (d). Scale bar 50µm.
