# Supplementary material for: Role of the receptor for advanced glycation endproducts (RAGE) in retinal vasodegenerative pathology during diabetes in mice
Source: Diabetologia. 2015 Feb 17;58(5):1129–37. doi: 10.1007/s00125-015-3523-x (PMC4392170; doi:10.1007/s00125-015-3523-x)
Supplement: Supplementary file 4 — (PDF 62 kb) [file 125_2015_3523_MOESM4_ESM.pdf]

ESM Table 1 Primer sequences used in qPCR analysis

Gene	Forward sequence	Reverse sequence
Rage	5′-TCAGGTCCACTGGATAAAGGAT-3′	5′-AGGTGCCCTCATCCTCGT-3′
Glo-1	5′-AAGAAGCCTGATGACGGGAA-3′	5′-GCTGTCCCCCATACCTCAAA-3′
β-Actin	5′-GATGCAGAAGGAGATTACTGC-3′	3′-CCACCGATCCACACAGAGTA-3′
